# A Thorough Examination of the Solution Conditions and the Use of Carbon Nanoparticles Made from Commercial Mesquite Charcoal as a Successful Sorbent for Water Remediation

**DOI:** 10.3390/nano13091485

**Published:** 2023-04-27

**Authors:** Tarig G. Ibrahim, Rasmiah S. Almufarij, Babiker Y. Abdulkhair, Rasha S. Ramadan, Mohamed S. Eltoum, Mohamed E. Abd Elaziz

**Affiliations:** 1 Chemistry Department, Faculty of Science, Sudan University of Science and Technology (SUST), Khartoum P.O. Box 13311, Sudan; 2 Department of Chemistry, College of Science, Princess Nourah Bint Abdulrahman University, P.O. Box 84428, Riyadh 11671, Saudi Arabia; 3 Chemistry Department, College of Science, Imam Mohammad Ibn Saud Islamic University (IMSIU), Riyadh 11564, Saudi Arabia; 4 Central Research Laboratory, Female Campus, King Saud University, Riyadh 11495, Saudi Arabia

**Keywords:** carbon nanoparticles, mesquite, water treatment, pharmaceutical pollutants, chlorohexidine

## Abstract

Water pollution has invaded seas, rivers, and tap water worldwide. This work employed commercial Mesquite charcoal as a low-cost precursor for fabricating Mesquite carbon nanoparticles (MUCNPs) using a ball-milling process. The scanning electron energy-dispersive microscopy results for MUCNPs revealed a particle size range of 52.4–75.0 nm. The particles were composed mainly of carbon with trace amounts of aluminum, potassium, calcium, titanium, and zinc. The X-ray diffraction peaks at 26.76 and 43.28 2θ° ascribed to the (002) and (100) planes indicated a crystalized graphite phase. Furthermore, the lack of FT-IR vibrations above 3000 cm^−1^ showed that the MUCNPs were not functionalized. The MUCNPs’ pore diameter, volume, and surface area were 114.5 Ǻ, 0.363 cm^3^ g^−1^, and 113.45 m^2^ g^−1^. The batch technique was utilized to investigate MUCNPs’ effectiveness in removing chlorohexidine gluconate (CHDNG) from water, which took 90 min to achieve equilibrium and had an adsorption capacity of 65.8 mg g^−1^. The adsorption of CHDNG followed pseudo-second-order kinetics, with the rate-limiting step being diffusion in the liquid film. The Langmuir isotherm dominated the CHDNG adsorption on the MUCNPs with a correlation coefficient of 0.99. The thermodynamic studies revealed that CHDNG adsorption onto the MUCNPs was exothermic and favorable, and its spontaneity increased inversely with CHDNG concentration. The ball-milling-made MUCNPs demonstrated consistent efficiency through regeneration–reuse cycles.

## 1. Introduction

Water is an essential life element, temperature buffering agent, and metabolism media for living organisms and human beings [1]. Knowing that water pollution by pharmaceutical products (PhPs) was discovered in 1960 but was not considered a threat until 1999 may help to understand the enormity of the problem [2,3]. Municipal and sewage waste, runoff from agricultural areas, and machine-washing in pharmaceutical enterprises are all significant sources of water contamination that provide intermediates, raw materials, and active pharmaceutical components (APIs) [4,5,6]. Additionally, transboundary pollution can affect areas thousands of miles from the emergence point, reaching other countries through oceans, and thus, making this a global concern [7,8,9]. Although chronic exposure to PhPs in water is considered a severe public health problem, conventional treatments have not been successful in keeping them out of oceans, rivers, or tap water [10]. Water pollution is considered to be a direct cause of cause for the spread of renal failure, cancers, congenital disabilities, and various diseases [11,12]. However, the PhPs are necessary for modern life, as exemplified by infectious diseases treatments and agricultural productivity, and demand continues to grow [13,14]. Hence, this problem is not one that can be prevented entirely because of the continuous emergence of contaminants; however, it could be reduced via appropriate treatments. Recently, the use of sterilizers has increased enormously because of the COVID-19 pandemic. Chlorhexidine-di-gluconate (CHDNG) is a cationic bisbiguanide, which has been used for over 50 years in sterilizers, wound dressing, and mouthwashes [15,16]. This API is a drug with a dose released in its entirety into the aquatic system [17,18]. CHDNG is environmentally hazardous and toxic to marine organisms and humans [19,20]. An additionally danger of anti-microorganism drugs occurring in water is the enhancement of anti-drug-resistant bacteria, which become immune and create new diseases via their mutation [21]. Despite its widespread application, relatively little research has focused on effectively eliminating these medicinal ingredients from water. Since PhPs retain their chemical structure and are biologically undegradable, it is necessary to search for viable solutions [22,23]. The incomplete oxidative degradation generates byproducts of more toxic products than CHDNG [24]. Hence, in the present research, adsorption is the process used for removing this pollutant from water. Compared to metal oxides, carbonaceous materials (CRMs) are compelling—they are preferable sorbents because of their high adsorptivity, low cost, insolubility in water, and environmentally friendly characteristics [25,26].

CMs are traditionally used as a color or odor remover due to their outstanding adsorbing capabilities [27]. In addition, their low-cost and excellent adsorbing properties made CMs allotropes excellent as potential water treatment substrates [28]. In contrast to metal oxides and clay, CMs are resistible to acidic and basic mediums [29]. In addition, they can be manufactured from various agricultural and industrial wastes, the disposal of which in this way is considered a solution to an environmental problem [30,31]. Among the CMs allotropes, CNPs are known for their high surface area through which the adsorption capacity may increase significantly. Because of the high expense of preparing carbon materials from virgin resources, which limits their practical application as a sorbent, it is preferable to produce carbonaceous material from refuse [32,33]. Consequently, researchers have prepared CMs from various agricultural scraps, such as lemonwood, orange peels, onion shells, and potato peels [34,35]. Additionally, CMs have been synthesized from edible oil industrial waste, such as sunflower and olive seeds [36,37]. The mesquite (MSQ) tree is spread across large regions of the USA, the Caribbean, the Middle East, and Africa [38,39]. In Texas, USA, 70 million hectares were reduced to 22 million usable hectares due to MSQ invasion. The Soil and Water Conservation Board of Texas State spent ten years and around fifty million USD to treat only 300,000 hectares with pesticides and mechanically remove of this invasive species [40]. The MSQ tree’s ability to enrich the soil around its roots makes it attractive for desertification combat, and it has been employed in arid African countries [41,42]. The MSQ tree is a severe soil-moisture competitor, its root system penetrating as deep as 53 m, which, when combined with its surface roots, covers a 15 m perimeter [43,44]. The rapid spread of MSQ trees in the United States and Sudan is proof of this. Herbicides applied topically or to the plant’s roots will not eradicate all instances of MSQ, and minimal herbicide harm can even stimulate new growth in the trees [45]. Because it is the most low-cost MSQ removal protocol, controlled burning has been used as a charcoal source in Sudan [46]. 

The aim of this work is to use the agricultural problem of MSQ trees as a commercially available source for producing carbon nanoparticles, which may result in achieving two goals in one. The characteristics of the fabricated MSQ carbon nanoparticles (MUCNPs) was investigated, and the sorbent was tested to remove antiseptic compounds, as exemplified by CHDNG.

## 2. Experiment

### 2.1. Materials

Commercial MSQ charcoal was purchased from a local market in Khartoum, Sudan. UniLab Pharmaceuticals, Mumbai, India, was the source of the CHDNG.

### 2.2. Preparation of MUCNPs Nanoparticles

Five grams of MC were crushed in a porcelain mortar and then moved to a stainless-steel milling crucible with a capacity of fifty milliliters. Each crucible contained seven stainless steel balls, and the ball-milling equipment was run at 500 RPM for 10.0 h. A powder was generated by sonicating 100 mL of distilled water (DW) for 4 h before being filtered and dried at 120 °C.

### 2.3. Characterization of MUCNPs

The prepared nanoparticles were analyzed using scanning electron energy-dispersive microscopy (SEM-EDX, JSM-IT500, MA, USA), powder X-ray (Bruker-D8 Advance-XRD, Billerica, MA, USA), Fourier-transform infrared spectroscopy (FTIR-Tracer-100, Shimadzu, Japan), and a surface analyzer (ASAP 2020 Micromeritics surface, Miami, FL, USA).

### 2.4. Adsorption Studies

The CHDNG Adsorption tests on the MUCNPs were studied via a batch technique. A mixture of 50 mg of MUCNPs and 120 mL of 100 mg L^−1^ CHDNG was stirred using a magnetic stirrer, and the data collected were utilized in the study of sorption kinetics. Additionally, CHDNG sorption onto MUCNPs was examined, along with the effect of pH within a range of pH 2 to pH 10. Fifty milligrams of SMCNPs were mixed with two hundred milliliters of the adjusted solution and stirred for two hours. Furthermore, sorption experiments were also performed at 20 °C using 25, 50, 75, and 100 mg L^−1^ of CHDNG concentrations to examine the effect of this variable, and the obtained results were employed in the kinetic investigations. The CHDNG removal from the four serial concentrations was tested within a 20 to 50 °C temperature range in order to study the effect of temperature.

### 2.5. Regeneration Study

Sorbent reusability is an economic factor; therefore, examining this factor is essential. Since the CHDNG is highly soluble in ethanol, the used sorbent was sonicated with 10 mL ethanol for 15.0 min, filtered, rinsed with another 10 mL ethanol, and dried at 120 °C for two hours.

## 3. Results and Discussions 

### 3.1. Characterizations

**Figure 1:**
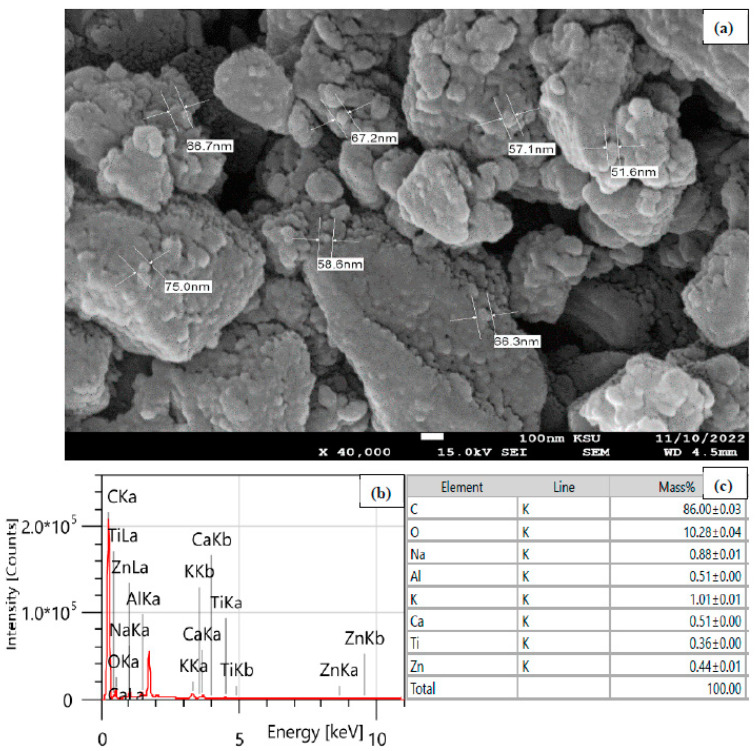
(**a**) SEM, (**b**) EDX spectrum, and (**c**) the elemental composition table of the MUCNPs.

The SEM examination was used to analyze the morphology of the MUCNPs’ surfaces. Figure 1a shows the SMCNPs SEM results, which possessed particles of sizes ranging from 52.4 to 75.0 nm. Additionally, the EDX was utilized to examine the elemental composition of the MUCNPs (Figure 1b,c). The commercial MC was composed mainly of carbon (86.0%) and trace amounts of aluminum, potassium, calcium, titanium, and zinc.

**Figure 2:**
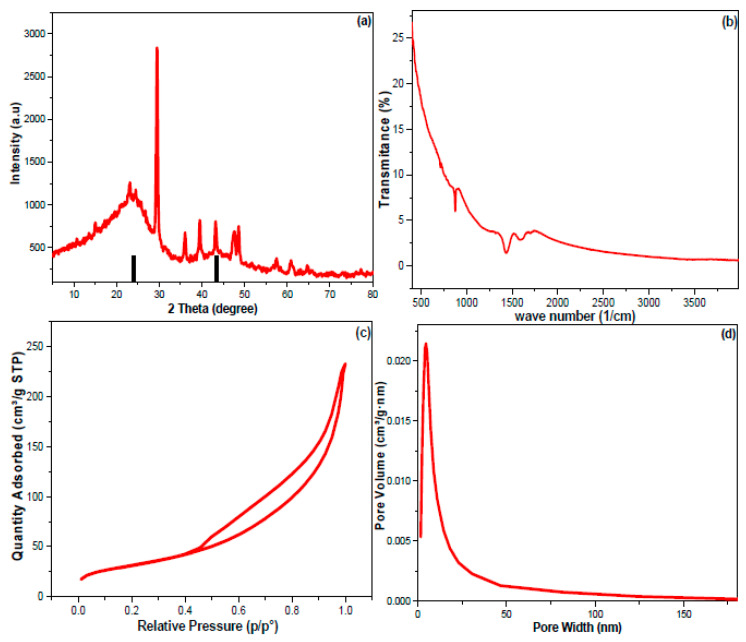
(**a**) XRD, (**b**) FT-IR, (**c**,**d**) the N_2_ adsorption–desorption results of the produced MUCNPs.

The MUCNPs’ crystallinity and phase purity were tested via the XRD (Figure 2a). The data obtained showed that there were diffraction peaks at 26.76 and 43.28 2-theta degrees that can be ascribed to the (002) and (100) planes of a cubical–lattice graphite phase [47]. The complexity of the XRD spectrum can be explained by the multi-element composition of the commercial muskat charcoal revealed by the EDX. 

Moreover, FTIR spectrophotometry was employed to survey the functional groups of the fabricated MUCNPs (Figure 2b). The results of MUCNPs revealed vibration peaks from 400 to 900 cm^−1^ corresponding to the metal oxides revealed by EDX. The bands at 1429, 1590, and 1708 cm^−1^ can be assigned to the MUCNPs’ carbon skeleton. Additionally, the absence of vibrations above 3000 cm^−1^ implied that the MUCNPs were not functionalized.

The N_2_ adsorption–desorption technique was used to study the surface properties of MUCNPs (Figure 2c,d). The MUCNPs have an H3 hysteresis loop, typical of cylindrical-pored mesoporous materials [48,49,50]. The Brunauer–Emmett–Teller (BET) method was selected to compute the MUCNPs’ surface area (SA), and the Barrett–Joyner–Halenda (BJH) method was chosen to estimate the MUCNPs’ pore diameter and volume (PD and PV). The MUCNPs possessed PD = 114.5 Ǻ, PV = 0.363 cm^3^ g^−1^, and SA = 113.45 m^2^ g^−1^. 

### 3.2. Adsorption of CHDNG

**Figure 3:**
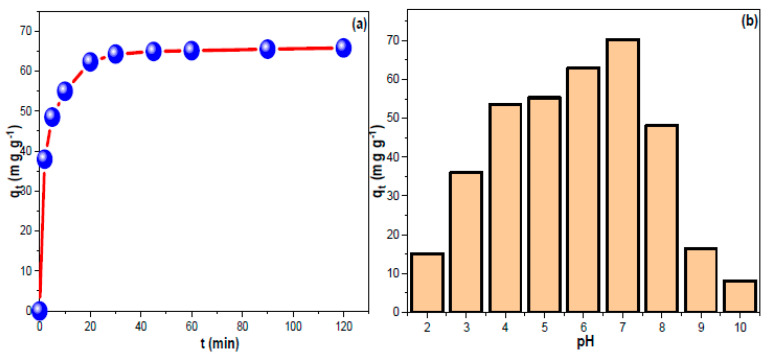
(**a**,**b**) The impact of contact time and the influence of pH on CHDNG sorption by MUCNPs.

Figure 3a depicts the contact time influence on CHDNG removal by MUCNPs. The removal trend progressed up to 90 min (equilibrium time), and the obtained experimental q_t_ value was 65.8 mg g^−1^. The temperature and initial feeding concentration are crucial factors affecting the adsorption process. Figure 3b demonstrates the proportionality of the resulting q_t_ and feeding concentration. In conclusion, a higher starting concentration has the potential to provide a powerful force that aids in the dispersal of contaminants. However, increasing the solution temperature decreased the CHDN elimination rate (exothermic sorption) [51]. In addition, the qt correlated positively with feed concentrations, suggesting that a 2:1 sorbent mass-to-solution ratio would be appropriate for the concentrations explored in this study. These results were comparable to current CMs in the literature regarding rapid absorption, short equilibrium duration, and high experimental q_t_ values [52,53,54,55,56]. Notably, the high qt from 100 mg L^−1^ CHDNG suggested the potential utility of SMCNPs in treating contaminants in significant concentrations. However, the fact that they could remove almost half of the amounts at 25 mg L^−1^ suggests they could be used effectively in water treatment systems where low quantities are predicted [57]. The concentration and temperature impacts on CHDNG removal through MUCNPs were investigated (Figure 3b). The inversed proportionality between temperature and CHDNG removal implied exothermic sorption [58]. The adsorption of CHDNG was studied, and its sensitivity to pH was determined (Figure 3b). The q_t_ data indicated that pH 7.0 was best for CHDNG sorption onto the MUCNPs. The CHDNG’s electron-rich sites may become protonated in low pH conditions. However, in an alkaline environment, the hydroxyl groups may deprotonate the CHDNG’s acidic sites and/or compete with the pollutants for the adsorption sites [59].

### 3.3. Kinetics

Equation (1) was used to determine the adsorption capacity, expressed as the milligrams of CHDNG that may be adsorbed onto one gram of MUCNPs (qt, in mg g^−1^). In order to investigate the adsorption rate, the pseudo-first-order (PF) and pseudo-second-order (PS) kinetic models (Equations (2) and (3)) were utilized. Furthermore, the liquid-film diffusion model (LD, Equation (4)) and the intraparticle diffusion model (ID, Equation (5)) were used in order to investigate the adsorption step that is present in adsorption [60,61].

(1) $$Q_{t}=\frac{\left(C_{o}-C_{t}\right)V}{m}$$

(2) $$ln\left(q_{e}-q_{t}\right)=\ln q_{e}-k_{1}\cdot t$$

(3) $$\frac{1}{q_{t}}=\frac{1}{k_{2}{\cdot q}_{e}^{2}t}+\frac{1}{q_{e}}$$

(4) $$q_{t}=K_{IP}*t^{\frac{1}{2}}+C_{i}$$

(5) $$ln(1-F)=-K_{LF}*t$$

**Figure 4:**
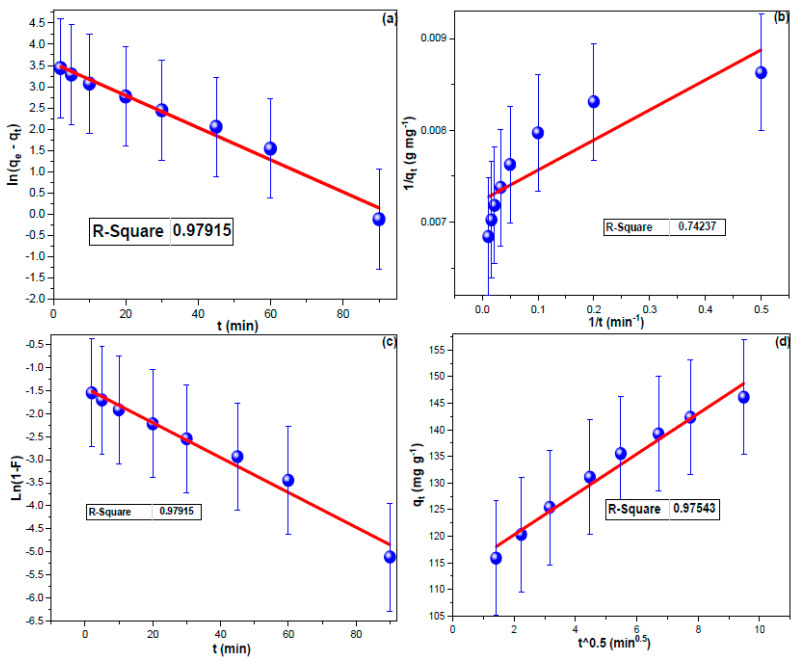
(**a**) PF, (**b**) PS, (**c**) LD, and (**d**) ID investigations for CHDNG sorption from 100 mL of 100 mg L^−1^ onto the Fabricated MUCNPs.

The equilibrium adsorption capacity is denoted by q_e_ (mg g^−1^), while the PF, PS, ID, and LD constants are represented by k_1_(min^−1^), k_2_(g mg^−1^ min^−1^), k_IP_ (mg g^−1^ min^−1/2^), and k_LF_ (min^−1^), respectively. The C_i_ (mg g^−1^) was the boundary layer factor [62]. The linear PF and PS plots for the elimination of CHDNG by MUCNPs are shown in Figure 4a,b. CHDNG adsorption on MUCNPs followed the PF kinetic model, with the LD mechanism influencing its sorption on MUCNPs (see Table 1). (Figure 4c,d). These findings supported the PF agreement that CHDNG adsorption depends primarily on migration from the solution to the MUCNP surface. However, the exceptionally high Ci value suggested that ID did not significantly regulate CHDNG sorption on MUCNPs [63]. The sorbent performance is excellent considering the CHDNG’s short uptake time onto MUCNPs and the experimental q_t_ value (Table 2).

### 3.4. Sorption Isotherms

**Figure 5:**
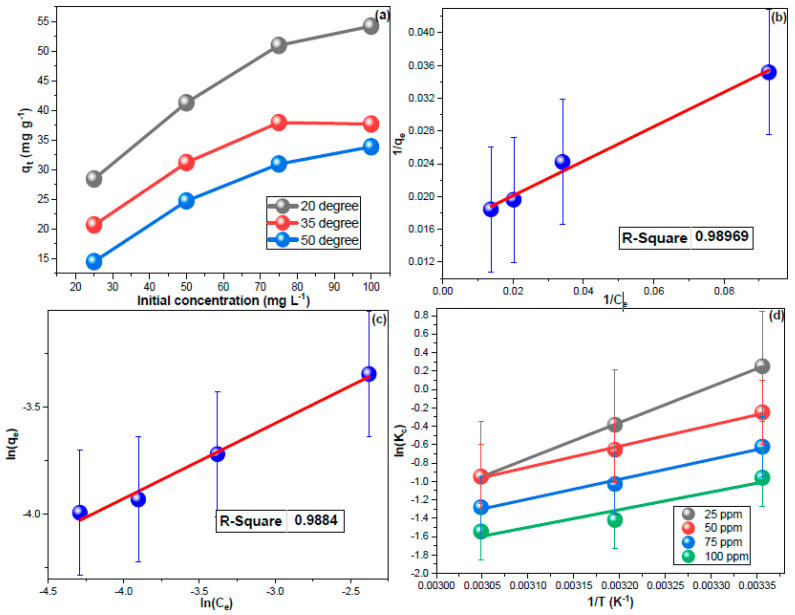
(**a**) The impact of temperature and fed CHDNG concentration, (**b**,**c**) LM and FM results, and (**d**) thermodynamic findings for CHDNG sorption onto MUCNPs.

The impact of CHDNG concentration on its removal by MUCNPs was investigated (Figure 5a). The experimental q_t_ increased proportionally as the feeding concentration increased, indicating that a 1:2 sorbent-to-solution ratio can effectively treat a 100 mg L^−1^ CHDNG pollution [59]. These obtained results implied the applicability of MUCNPs to remediate industrial wastewater and contaminated water resources. Further, the results of sorption equilibria from different CHDNG concentrations were utilized for the adsorption isotherm investigations. The adsorption isotherms have been studied using the Langmuir (LM, Equation (6)) and Freundlich (FM, Equation (7)) as the most widely applied isotherm models.

(6) $$\frac{1}{q_{e}}=\frac{1}{K_{L}q_{m}}.\frac{1}{C_{e}}+\frac{1}{K_{L}}$$

(7) $$\ln q_{e}=\ln K_{F}+\frac{1}{n}\ln C_{e}$$

The LM and FM were K_L_ (L mg^−1^) and K_F_ (L mg^−1^).

Ce (mg L^−1^) is the CHDNG equilibrium concentration,

Q_m_ (mg g ^−1^) is the maximum q_t_, and

n (arbitrary) is the Freundlich-heterogeneity factor.

Figure 5b,c depicts the isotherms plots, and their computed results are shown in Table 3. Removing CHDNG through MUCNPs functioned better using LM, and the 1/n value being more than unity indicated the unfavorability of multilayer sorption (Table 3) [70,71,72,73].

### 3.5. Sorption Thermodynamics

The influence of medium temperature on the CHDNG adsorption was explored using different concentrations. Figure 5a revealed a reverse proportionality between qt and temperature, which implied exothermic sorption. Furthermore, the temperature impact results were employed to analyze sorption thermodynamics (Figure 5d). The entropy (ΔS°) and enthalpy (ΔH°) were determined using Equation (8), and then the Gibbs free energy (ΔG°) was computed using their values in Equation (9). The resulting values are tabulated in Table 3.

(8) $$\ln K_{c}=\frac{\Delta H^{o}}{RT}+\frac{\Delta S^{o}}{R}$$

(9) $$\Delta G^{o}=\Delta H^{o}-T\Delta S^{o}$$

Exothermic sorption was predicted by the negative ΔH° values within the tested concentrations (Table 3). The negative sign of ΔS° values may indicate the spontaneity of CHDNG removal through MUCNPs. Additionally, the decrease in ΔG° values inversely with temperature corroborates the exothermic discovery of this sorption [74,75,76,77]. The use of this sorbent for water treatment is encouraged by the inverse proportionately of ΔG° with the concentration. Additionally, the ΔH° values of less than 80 kJ mol^−1^ can be used to forecast the physisorption nature of this process.

### 3.6. Regeneration–Reuse Investigations

**Figure 6:**
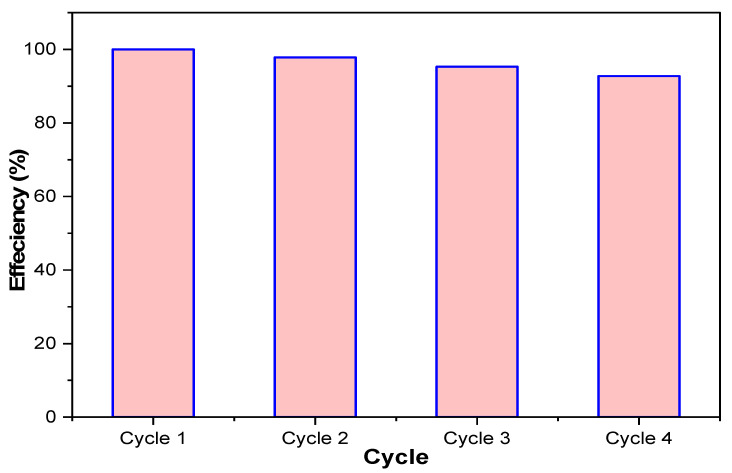
The reuse performance of the prepared MUCNPs for removing CHDNG from aqueous solutions.

The MUCNPs sorbent was regenerated in four consecutive batches. The efficiency of the regenerated MUCNPs was examined by mixing 50 mg of sorbent with 120 mL CHDNG solution (Figure 6). The MUCNPs showed an average removal percentage of 97.4% during the four cycles with an RSD of 2.6%. The excellent performance of regenerated MUCNPs can be attributed to the nature of the physisorption, with the sorption being controlled by LD and the one-layer sorption trend (LM).

## 4. Conclusions

Commercial MSQ charcoal was employed as a low-cost precursor for fabricating MUCNPs via a ball-milling process. SEM-EDX, FTIR, BET, and XRD were utilized to characterize the produced MUCNPs. The batch protocol was employed to study the removal of CHDNG from water. The removal process progressed for one and a half hours, after which there was no significant progress, and a q_t_ of 65.8 mg g^−1^ was attained. The kinetic investigations showed that the CHDNG adsorption followed the PS kinetic model, and LD influenced CHDNG adsorptions onto MUCNPs. The CHDNG adsorption on the MUCNPs fitted the LM with a correlation coefficient of 0.99. The ball-milling-made MUCNPs performed excellently in removing CHDNG during the regeneration–reuse cycles.

## Figures and Tables

**Table 1:** The kinetics parameters of CHDNG sorption on MUCNPs from the 100 mg L^−1^ solution.

Adsorption kinetic						
Adsorption rate order						
qe exp. (mg g^−1^)	PF			PS		
	qe cal. (mg g^−1^)	R^2^	k_1_	qe cal. (mg g^−1^)	R^2^	k_2_
65.771	6.644	0.888	0.051	66.031	0.979	0.010
Adsorption mechanism						
LD			ID			
K_LF_ (min^–1^)	R^2^		K_IP_ (mg g^−1^ min^0.5^)		C (mg g^−1^)	R^2^
0.066	0.888		4.006		38.979	0.733

**Table 2:** Comparing the MUCNP’s performance in removing CHDNG with literature findings.

Sorbent	Experimental Adsorption Capacity (mg g^−1^)	Reference
MUCNPs	65.8	This study
Acid-modified fly ash	23.6	[17]
Acid-functionalized activated carbon	25.0	[64]
Cellulosic fibers	27.5	[65]
Granular activated carbon	19.0	[66]
HF-functionalized activated carbon	24.0	[67]
HCl-functionalized activated carbon	23.5	[68]
Granular activated carbon	17.5	[69]

**Table 3:** The isotherm and thermodynamic findings of the CHDNG sorption onto MUCNPs.

Adsorption Isotherms						
LM				FM		
R^2^	qm (mg g^−1^)	KL (L mg^−1^)		R^2^	K_f_ (L mg^−1^)	n^−1^ (a.u.)
0.990	62.872	0.076		0.988	0.080	2.856
Thermodynamic parameters						
Fed conc. (mg L^−1^)	ΔH^°^ (kJ mol^−1^)	ΔS^°^ (kJ mol^−1^)	ΔG^°^ (kJ mol^−1^) 298 K	ΔG^°^ (kJ mol^−1^) 313 K		ΔG^°^ (kJ mol^−1^) 328 K
25	−32.518	−0.107	−0.615	0.991		2.597
50	−18.953	−0.066	0.651	1.638		2.625
75	−17.875	−0.065	1.592	2.572		3.552
100	−15.981	−0.062	2.499	3.429		4.360

## Data Availability

All data is available under reasonable request from the corresponding author.
